# Acknowledgment to the Reviewers of *Tomography* in 2022

**DOI:** 10.3390/tomography9010014

**Published:** 2023-01-20

**Authors:** Tomography Editorial Office

**Affiliations:** MDPI AG, St. Alban-Anlage 66, 4052 Basel, Switzerland

High-quality academic publishing is built on rigorous peer review. *Tomography* was able to uphold its high standards for published papers due to the outstanding efforts of our reviewers. Thanks to the efforts of our reviewers in 2022, the median time to first decision was 26 days and the median time to publication was 48 days. Regardless of whether the articles they examined were ultimately published, the editors would like to express their appreciation and thank the following reviewers for the time and dedication that they have shown *Tomography*:
Abondio, PaoloMascia, GiuseppeAbu-Omar, AhmadMaselli, FilippoÁcs, PéterMatveeva, MariaAfat, SaifMazzucco, WalterAhmadi, AmirmasoudMcGowan, DanielAhmed, JunaidMejzlik, JanAkiyama, HironoriMenéndez-Menéndez, JavierAkkouch, AdilMichalak, EwaAndresciani, FlavioMilitello, CarmeloAngelopoulou, GeorgiaMinea, RaduAnkrah, Alfred O.Minini, AndreaAprile, CarloMoffat, Bradford AArdenkjær-Larsen, Jan HenrikMohamed, IslamAschauer, Manuela AdelindeMolnár, ViktorAtaeinia, BaharMoon, EjungBadari, AmbugaMoran, LuzBalestra, CostantinoMorasiewicz, PiotrBarile, AntonioMourad, Pierre D.Barton, Gregory P.Müller, LukasBartosz, GrzegorzNagel, SebastianBeer, MeinradNajafzadeh, EbrahimBelousov, Alexandr V.Nardi, CosimoBender, MichaelNardone, ValerioBertelli, ElenaNaselli, AngeloBesser, GeroldNaumova, Anna V.Bianco, FrancescoNemeth, ZsoltBjarnason, HaraldurNesteruk, Konrad P.Blocker, Stephanie J.NG, Curtise Kin CheungBonacchi, MassimoNicola, MarzilianoBorgheresi, AlessandraNijkamp, JasperBranca, Jacopo Junio ValerioNikaki, AlexandraBratu, MateiNordstrom, RobertBrommeland, TorNzwalo, HipolitoBruzzaniti, PlacidoObuchowicz, RafałBudzevich, MikalaiOh, Se-HongBuljevic, SuncicaOhno, NaokiBulum, AntonioOllivier, LucCamacho-Gutiérrez, Stephanie VirginiaOrso, DanieleCantisani, VitoOsipov, S. P.Carr, ShamusOsipov, Sergey PavlovichCassano, EnricoOsman, HamidChaichana, ThanapongOwda, Amani YousefChang, Ke-VinÖzmen, TolgaChassagne, FanetteÖztürk, ŞabanChaudhari, MeenalPanetta, DanieleChauhan, MunishPaone, GaetanoChavan, Sandeep KumarPapageorgiou, Ismini EChen, HaoPappas, EleftheriosChen, MingyiParaskevaidis, IoannisCheung, James Chung WaiPark, IlwooCho, SeungryongParoder, ViktoriyaChou, Shah-HwaParuccini, NicolettaChou, Yu-ChengPashazadeh, AliCiolac, DumitruPattichis, MariosClaps, FrancescoPaul-Andrei, StefanCorell, AlbaPedro, Ana Sofia PereiraCosta, Andre LuizPérez, WilliamCosta, PedroPerez-Berna, Ana JoaquinaCostagli, MauroPeriakaruppan, RajivCross, Chad L.Pesce, AlessandroCsecs, IbolyaPetoukhova, AnnaDamásio, JoanaPires, Ivan MiguelDatta, ArkaPleass, HenryDe Alcantara Camejo, FlavioPoje, GorazdDe Paoli, AntoninoPosa, AlessandroDe Paolis, MassimilianoPozzan, RiccardoDe Riese, Werner T.W.Pozzessere, ChiaraDean, MarcPrandoni, PaoloDekeyzer, SvenPrezoto, Benedito C.Deng, HuaifuPyrgelis, Efstratios-StylianosDespande, AparnaQian, WeiDevane, MichaelQvist, NielsDi Domenico, GiovanniRakhmangulov, AleksandrDimov, AlexeyRavichandran, KollarigowdaDubinský, PavolRavichandran, Naresh KumarDurán-Poveda, ManuelRengaraj, ArunkumarDzierżak, RóżaRenzulli, MatteoEddy, RachelRizuana, IHEgger, JanRizzo, AlessandroElmi-Terander, AdrianRocha, Mateus GarciaEresen, AydinRodríguez-Reséndiz, JuvenalFaggioni, LorenzoRoi, CiprianFelippini, Ana Luiza De CarvalhoRothluebbers, SvenFermi, MatteoRoudenko, AlexandraFigueiredo, CarlosRuaro, BarbaraFiscaletti, MartaRusu, Mugurel ConstantinFlegar, LukaRymarczyk, TomaszFogante, MarcoRzepka, ZuzannaFoti, GiovanniSaenko, KateFragomeni, GionataSakai, MakotoFrandon, JulienSakamoto, ShinichiFu, ShengSakuramachi, MadokaFuss, Martina C.Salazar, AddissonGagliardo, CesareSantos, MiltonGallo, SalvatoreSarecka-Hujar, BeataGarcia Cabrera, EmilioSchiattarella, AntonioGarcía-Horsman, J.A.Schramm, SeverinGerke, OkeSchuetz, GunnarGermanese, DanilaSchweizer, BerndGhezzi, MicheleSconfienza, ElisaGiacomelli, LauraŠebek, JanGiordano, AntonioSeo, Bo KyoungGiovanni, DamianiSerikov, ArkadyGołąb, AdamSethi, GautamGontarz, MichałShaikh, Sadiya BiGonzález-Cortés, J.H.Shams, Abdullah BinGreco, TommasoShao, WeiGrist, JamesShim, YunGudey, Shyam KumarShin, TaehoonHägerling, RenéShiwarski, Daniel J.Hall Barbosa, Carlos RobertoShukla Dave, AmitaHamamoto, RyujiShukla, DivanshuHansen, Søren BaarsgaardSilva, HenriqueHarada, YukoSingh, KavitaHashimoto, YukiSkoczyński, SzymonHassani, BertrandSlavkova, Kalina PoletHayward, Mary-KateSorace, Anna G.Heid, IrinaSpinnato, PaoloIm, Jung-GiSpoerl, SteffenIshikawa, TetsuyaStanzione, ArnaldoIso-Mustajärvi, MattiStock, UlrichJaber, José RaduanStrzelecki, MichałJain, KartikSu, ShangJayaprakasam, Vetri SudarSu, YujieJensen-Kondering, UlfSurov, AlexeyJin, DiSvahn, Tony MartinJoshi, Pushpa RajSykaras, Alexandros G.Joshi, SameerTagliati, CorradoKallergi, MariaTama, Bayu AdhiKalogeropoulos, AndreasTamaskovics, BálintKalva, SanjeevaTamburrini, StefaniaKaraca, YelizTanwar, SwatiKassani, Sara H.Tarasconi, AntonioKelly, CliveTari, Daniele UgoKhan, Mohd ParvezTha, KhinKim, HarrisonTomić, JosipKlontzas, Michail E.Tun, Han NaungKolimas, ŁukaszUrbschat, Steffi M.Komatsu, TeruyaVara, GiulioKommaraju, Sai ShilpaVarone, GiuseppeKoni, EndrinVerdolotti, TommasoKono, Atsushi K.Vergados, AthanasiosKopka, KlausVersari, AnnibaleKozminski, PrzemysławVetrano, Ignazio GaspareKromrey, Marie-LuiseVirgilli, EnricoKrzyżak, Artur TadeuszVirostko, JohnKumar, AmitWalocha, JerzyKumar, AshishWang, CunchuanKumar, ManojWang, JingyuKumari, AnjuWang, Myeong-HyeonKwok, EdmundWang, ShidanLandeiro, José AlbertoWelton, ThomasLavender, AndrewWilliams, JamesLeboucher, JulienWillmering, MatthewLee, HyunyeolWirestam, RonnieLee, Won WooWood, SossenaLee, Yen ChienWu, ChengyueLeng, XiandongWu, ChuanchengLepic, MilanXi, JinxiangLi, WeilianYadav, AmarishLiang, ZifeiYamada, KenichiLippolis, Piero VincenzoYamaguchi, MitsutakaLiu, XiujianYeh, Sheng-LihLjungberg, EmilYoshiura, TakashiLong, Alexander M.Yu, ZidanLuchian, IonutYuan, ZhenLukosevicius, SauliusZabulis, XenophonLy, Kim HeangZapata, UrielLyrer, PhilippeZedda, MarcosMa, LingZegrean, MihaelaMaccagnano, GiuseppeZhang, Han YuMacri, FrancescoZhang, WeiMaegerlein, ChristianZhang, WenxuMalek, LukaszZhao, BoMallio, Carlo AugustoZhao, ChonghangMałyszek-Tumidajewicz, JustynaZheng, XiaomingMancini, MariangelaZhou, MingliangMarchiano, Alfonso VittorioZhou, Yu-PengMarcu, LoredanaZöllner, Helge JörnMarra, PaoloZubair, MuhammadMartins, Jorge N.R.Zuin, Marco


